# Supplementary material for: Vegetation change over seven years in the largest protected Pacific Northwest Bunchgrass Prairie remnant
Source: PLoS One. 2020 Jan 24;15(1):e0227337. doi: 10.1371/journal.pone.0227337 (PMC6980589; doi:10.1371/journal.pone.0227337)
Supplement: Attachment — Submitted filename: PONE-D-19-27436_reviewX.pdf [file pone.0227337.s001.pdf]

PLOS ONE

Vegetation change over seven years in the largest protected Pacific Northwest Bunchgrass Prairie remnant.

--Manuscript Draft--

**Manuscript Number:**	PONE-D-19-27436
**Article Type:**	Research Article
**Full Title:**	Vegetation change over seven years in the largest protected Pacific Northwest Bunchgrass Prairie remnant.
**Short Title:**	Grassland vegetation change
**Corresponding Author:**	Joshua Patrick Averett, M.S.<br>Oregon State University<br>Union, OR UNITED STATES
**Keywords:**	Grassland vegetation change, plant invasion, plant community change, old fields, Palouse Prairie
**Abstract:**	Temperate grasslands are one of the most altered ecosystems on Earth. Consequently, conservation of important characteristics of such ecosystems (e.g. biodiversity) is uncertain even within grasslands that have been protected. Invasion by non-native plants is considered a primary threat to intact grasslands. Here, we evaluated native and non-native vegetation composition change over seven years in the largest Pacific Northwest Bunchgrass remnant. We sampled 124 permanent plots across the Zumwalt Prairie Preserve (northeastern Oregon, USA) twice, seven years apart. With data collected from three grassland community types (xeric prairie, mesic prairie, old fields), we asked: 1) how has species composition changed over time; (2) which species showed the greatest changes in abundance; and (3) how did abundance of Ventenata dubia (the most abundant non-native species) relate to patterns of native and non-native plant abundance? Vegetation composition changed in all three plant communities. Ventenata dubia, an annual non-native grass: 1) became the third most dominant species across the study area; 2) was the only non-native that increased in abundance substantially in all three communities; and 3) was negatively related to native perennial forb cover. Relative cover of non-native species decreased in old fields concomitant with increases in native bunchgrass (Festuca idahoensis) and V. dubia cover. Increased cover of native perennial grasses and non-native annual grasses in old fields were associated with loss of bare ground, but not with reductions in non-native perennial grass cover. Native species dominated in the mesic prairie; however, non-native cover (particularly V. dubia) increased (mean cover increased from 3 to 10%) while mean native perennial forb cover decreased (from 30 to 25%) over time. Continued shifts towards non-native annual grass dominance coupled with potentially declining native perennial forbs, may challenge conservation efforts in one of the last large tracts of Pacific Northwest Bunchgrass Prairie.
**Order of Authors:**	Joshua P. Averett, M.S.
	Lesley R. Morris
	Bridgett J. Naylor
	Robert V. Taylor
	Bryan A. Endress
**Additional Information:**	
**Question**	**Response**
**Financial Disclosure**<br><br>Enter a financial disclosure statement that describes the sources of funding for the work included in this submission. Review	Funding was provided by USDA NRI Competitive Grant Agreement No. 2006-35320-17244 (BE), USDA Forest Service Pacific Northwest Research Station # 05-JV-11261967-069 (BE), the Oregon State University Agricultural Research Foundation (LM, BE), and the Oregon State University Agriculture Experiment Station (LM, BE). The funders had no role in study design, data collection and analysis, decision to

Powered by Editorial Manager® and ProduXion Manager® from Aries Systems Corporation

the submission guidelines for detailed requirements. View published research articles from *PLOS ONE* for specific examples.<br><br>This statement is required for submission and **will appear in the published article** if the submission is accepted. Please make sure it is accurate.<br><br>**Unfunded studies**<br>Enter: *The author(s) received no specific funding for this work.*<br><br>**Funded studies**<br>Enter a statement with the following details:<br>• Initials of the authors who received each award<br>• Grant numbers awarded to each author<br>• The full name of each funder<br>• URL of each funder website<br>• Did the sponsors or funders play any role in the study design, data collection and analysis, decision to publish, or preparation of the manuscript?<br>• **NO** - Include this sentence at the end of your statement: *The funders had no role in study design, data collection and analysis, decision to publish, or preparation of the manuscript.*<br>• **YES** - Specify the role(s) played.<br><br>* typeset	publish, or preparation of the manuscript.
**Competing Interests**<br><br>Use the instructions below to enter a competing interest statement for this submission. On behalf of all authors, disclose any competing interests that could be perceived to bias this work—acknowledging all financial support and any other relevant financial or non-financial competing interests.<br><br>This statement **will appear in the published article** if the submission is accepted. Please make sure it is accurate. View published research articles from *PLOS ONE* for specific examples.	The authors have declared that no competing interests exist.

Powered by Editorial Manager® and ProduXion Manager® from Aries Systems Corporation

**NO authors have competing interests**<br><br>Enter: *The authors have declared that no competing interests exist.*<br><br>**Authors with competing interests**<br><br>Enter competing interest details beginning with this statement:<br><br>*I have read the journal's policy and the authors of this manuscript have the following competing interests: [insert competing interests here]*<br><br>* typeset	
**Ethics Statement**<br><br>Enter an ethics statement for this submission. This statement is required if the study involved:<br><br>• Human participants<br>• Human specimens or tissue<br>• Vertebrate animals or cephalopods<br>• Vertebrate embryos or tissues<br>• Field research<br><br>Write "N/A" if the submission does not require an ethics statement.<br><br>General guidance is provided below. Consult the submission guidelines for detailed instructions. **Make sure that all information entered here is included in the Methods section of the manuscript.**	Field sampling took place on private lands within the ZPP. Permission for sampling was granted by and in collaboration with the landowner, The Nature Conservancy. Field sampling for this study was non-destructive, and did not harm endangered or protected species.

Powered by Editorial Manager® and ProduXion Manager® from Aries Systems Corporation

**Format for specific study types**<br><br>**Human Subject Research (involving human participants and/or tissue)**<br>• Give the name of the institutional review board or ethics committee that approved the study<br>• Include the approval number and/or a statement indicating approval of this research<br>• Indicate the form of consent obtained (written/oral) or the reason that consent was not obtained (e.g. the data were analyzed anonymously)<br><br>**Animal Research (involving vertebrate animals, embryos or tissues)**<br>• Provide the name of the Institutional Animal Care and Use Committee (IACUC) or other relevant ethics board that reviewed the study protocol, and indicate whether they approved this research or granted a formal waiver of ethical approval<br>• Include an approval number if one was obtained<br>• If the study involved *non-human primates*, add *additional details* about animal welfare and steps taken to ameliorate suffering<br>• If anesthesia, euthanasia, or any kind of animal sacrifice is part of the study, include briefly which substances and/or methods were applied<br><br>**Field Research**<br><br>Include the following details if this study involves the collection of plant, animal, or other materials from a natural setting:<br>• Field permit number<br>• Name of the institution or relevant body that granted permission	
**Data Availability**<br><br>Authors are required to make all data underlying the findings described fully available, without restriction, and from the time of publication. PLOS allows rare exceptions to address legal and ethical concerns. See the PLOS Data Policy and FAQ for detailed information.	Yes - all data are fully available without restriction

A Data Availability Statement describing where the data can be found is required at submission. Your answers to this question constitute the Data Availability Statement and **will be published in the article**, if accepted.<br><br>**Important:** Stating 'data available on request from the author' is not sufficient. If your data are only available upon request, select 'No' for the first question and explain your exceptional situation in the text box.<br><br>Do the authors confirm that all data underlying the findings described in their manuscript are fully available without restriction?	
**Describe where the data may be found in full sentences. If you are copying our sample text, replace any instances of XXX with the appropriate details.**<br><br>• If the data are **held or will be held in a public repository**, include URLs, accession numbers or DOIs. If this information will only be available after acceptance, indicate this by ticking the box below. For example: *All XXX files are available from the XXX database (accession number(s) XXX, XXX.*).<br>• If the data are all contained **within the manuscript and/or Supporting Information files**, enter the following: *All relevant data are within the manuscript and its Supporting Information files.*<br>• If neither of these applies but you are able to provide **details of access elsewhere**, with or without limitations, please do so. For example:<br><br>*Data cannot be shared publicly because of [XXX]. Data are available from the XXX Institutional Data Access / Ethics Committee (contact via XXX) for researchers who meet the criteria for access to confidential data.*<br><br>*The data underlying the results presented in the study are available from (include the name of the third party*	All data for this manuscript have been submitted to a data journal 'Data In Brief' where all raw data and accompanying figures and tables will be available.

and contact information or URL).<br>• This text is appropriate if the data are owned by a third party and authors do not have permission to share the data.<br><br>* typeset	
Additional data availability information:	

Powered by Editorial Manager® and ProduXion Manager® from Aries Systems Corporation

Manuscript
Click here to download Manuscript Averett_etal_ZumwaltVegChange.docx

Title: Vegetation change over seven years in the largest protected Pacific Northwest Bunchgrass Prairie remnant.

Running head: Grassland vegetation change

Authors: Joshua P. Averett[1], Lesley R. Morris[1], Bridgett J. Naylor[2], Robert V. Taylor[3], and Bryan A. Endress[1]

Author addresses: [1]**Averett JP** (averettj@oregonstate.edu), **Morris LR** (lesley.morris@oregonstate.edu), **Endress BA** (bryan.endress@oregonstate.edu), Eastern Oregon Agricultural Research Center, Oregon State University, Union, OR 97883, USA.

[2]**Naylor BJ** (bridgett.naylor@usda.gov), USDA Forest Service, Pacific Northwest Research Station, 1401 Gekeler Ln., La Grande, OR, 97883, USA.

[3]**Taylor RV** (robert.v.taylor@outlook.com), National Wildlife Refuge Association, Enterprise, Oregon, 97828, USA.

Author contributions: BE, LM, JA, RT, and BN conceived and designed the research; JA, BE, LM, BN, and RT performed the experiment, analyzed the data, and wrote the edited manuscript.

* Corresponding author
E-mail: averettj@ oregonstate.edu

Abstract

Temperate grasslands are one of the most altered ecosystems on Earth. Consequently, conservation of important characteristics of such ecosystems (e.g. biodiversity) is uncertain even within grasslands that have been protected. Invasion by non-native plants is considered a primary threat to intact grasslands. Here, we evaluated native and non-native vegetation composition change over seven years in the largest Pacific Northwest Bunchgrass remnant. We sampled 124 permanent plots across the Zumwalt Prairie Preserve (northeastern Oregon, USA) twice, seven years apart. With data collected from three grassland community types (xeric prairie, mesic prairie, old fields), we asked: 1) how has species composition changed over time; (2) which species showed the greatest changes in abundance; and (3) how did abundance of *Ventenata dubia* (the most abundant non-native species) relate to patterns of native and non-native plant abundance? Vegetation composition changed in all three plant communities. *Ventenata dubia*, an annual non-native grass: 1) became the third most dominant species across the study area; 2) was the only non-native that increased in abundance substantially in all three communities; and 3) was negatively related to native perennial forb cover. Relative cover of non-native species decreased in old fields concomitant with increases in native bunchgrass (*Festuca idahoensis*) and *V. dubia* cover. Increased cover of native perennial grasses and non-native annual grasses in old fields were associated with loss of bare ground, but not with reductions in non-native perennial grass cover. Native species dominated in the mesic prairie; however, non-native cover (particularly *V. dubia*) increased (mean cover increased from 3 to 10%) while mean native perennial forb cover decreased (from 30 to 25%) over time. Continued shifts towards non-native annual grass dominance coupled with potentially declining native

perennial forbs, may challenge conservation efforts in one of the last large tracts of Pacific Northwest Bunchgrass Prairie.

KEY WORDS: Plant invasion; old fields; Plant community change; Annual grass invasion; Palouse Prairie; *Ventenata dubia*

Introduction

Temperate grasslands are among the most threatened biomes on Earth and are the most altered ecosystems in North America [1]. Only about 1% of North America's temperate grasslands are in a relatively natural state, and even those that remain have been subjected to significant anthropogenic stressors, e.g., widespread livestock grazing, intentional and accidental introduction of non-native species, and localized settlement/cultivation and subsequent abandonment [1-2]. Because of widespread alteration to temperate grasslands in North America, the ability of these systems to maintain the ecological services, such as biodiversity, that they have historically provided is uncertain [1,3]. Invasion by non-native plant species has been identified as one of the most serious threats to ecosystem function and native biodiversity in remaining temperate grassland areas [4]. Non-native plant invasions have altered grassland structure, reduced floral and faunal diversity, altered disturbance regimes, and, in some circumstances have resulted in complete shifts in species composition and ecosystem function [5-6]. Although plant invasions, to date, have not resulted in species extinctions [7], they may ultimately lead to the loss of species over time [8]. Identification of invading non-natives plants and the dynamics of their spread including associations with native vegetation can provide critical information for the prioritization of conservation, research and monitoring efforts,

evaluation of the potential to control and/or adapt to invasions, and understanding the potential impacts of invasion on native communities.

The Zumwalt Prairie Preserve (hereafter ZPP), the focus of this study, encompasses around 13,350 ha in northeastern Oregon, USA, and is located within the largest remaining intact Pacific Northwest Bunchgrass Prairie (the larger Zumwalt Prairie, ~ 65,000 ha). The Pacific Northwest Bunchgrass Prairie (hereafter PNB), once occupied ~ 8 million ha of Oregon, Washington, Idaho and portions of Western Canada [9-11]. It is also one of the least described and least studied of the major vegetation types in North America despite the endangered status for portions of the PNB [9,12]. Consequently, the ZPP was purchased by The Nature Conservancy (hereafter TNC) in two land acquisitions (~ 11,000 ha in 2000 and ~ 2,400 ha in 2006) and is managed to support the conservation of habitat for native species including the largest known population of the threatened Spalding's catchfly (*Silene spaldingii;* [13]) as well as diverse native forb (112 species; ~64% of vascular plant species; [14, 15]) and bee (more than 200 species in 27 genera; [15]) communities. Though protected, the ZPP is subject to anthropogenic stressors that are considered threats to conservation of native temperate grasslands (e.g., legacy effects of past cultivation, livestock grazing, invasion by non-native plants; [1,4]). Past research has evaluated the effects of cattle grazing on vegetation structure, and bird and bee habitat requirements [15-16], the potential to restore old fields that had been abandoned following cultivation [17-18], and impacts of non-native plants on passerine birds [19].

Invasion by non-native plant species, although a recognized threat to the PNB, has been studied less than other disturbances on the PNB. Two studies have documented an increase in *Ventenata dubia* in PNB canyon grasslands since the 1980s and dominance by the invasive annual grass in soil seed banks of old fields [20-21]. A previous evaluation (2008 and 2009) of

non-native plant distributions conducted within the upland prairie portion (~ 5,100 ha) – excluding canyons– of the ZPP revealed that non-native grass species were widespread and among the most dominant vascular plant species across the ZPP by 2008 [22]. The most abundant non-native species were *Ventenata dubia*, *Poa pratensis* and annual *Bromus* species (*Bromus* spp; comprised of *Bromus arvensis* and *Bromus hordeaceous*) which had the fourth, fifth, and seventh highest total foliar cover respectively of all species across the study area [22]. Endress et al. (in Press [22]) described three broad plant communities within ZPP where non-native composition varied substantially. The communities identified (xeric prairie, mesic prairie, old fields) aligned well with other bunchgrass plant associations developed for the region that co-vary primarily with gradients in soil depth and water availability [9,23-24]. Non-native annual grasses (*Bromus* spp. and *Ventenata dubia*) were most abundant in the xeric prairie (highest heat load and greatest soil moisture deficit) associated with increased dominance of the native bunchgrasses *Poa secunda*, *Pseudoroegneria spicata* and *Danthonia unispicata*, and were least abundant in the wettest/deepest soil sites (mesic prairie, and old field communities; [22]). This pattern of apparently low invasion resistance of the more xeric sites to non-native annual grass invasion was consistent with previous observations [23-24] in the region. Non-native perennial grasses (e.g. *Poa pratensis* and *Thinopyrum intermedium*) were dominant in or near sites that had been previously cultivated (e.g., old fields). The detection of less invaded mesic communities coexisting alongside annual grass invaded xeric prairie and non-native perennial grass dominated old fields may suggest that never-plowed mesic prairie communities are more resistant to invasion or, alternatively, that invasion by non-native grasses from either the drier or wetter communities is inevitable [22]. The differences in non-native plant composition and abundance across community types in the ZPP suggest that some communities may be more susceptible to

invasion and alteration of species composition over time, while other communities may be more resistant to change. There are currently no studies that address vegetation change at a large scale over time in this system.

Our objectives were to analyze vegetation composition change over seven years across the ZPP. For this, we resampled (in 2015 and 2016 – seven years later) permanent plots that were established and initially sampled in 2008 and 2009 within the three community types described by Endress et al. (in Press [22]). We addressed the following questions: (1) how did species composition change over time in the three plant communities; (2) which species showed the greatest changes in abundance; and (3) how did abundance of *V. dubia* (most abundant non-native species) relate to the abundance of native perennial forbs, native perennial grasses, non-native perennial grasses, and other non-native annual grasses.

Methods

Field sampling took place on private lands within the ZPP. Permission for sampling was granted by and in collaboration with the landowner, The Nature Conservancy. Field sampling for this study was non-destructive, and did not harm endangered or protected species.

Study site

The ZPP is located in northeastern Oregon (45.577745 N 116.971754 W). Elevations range from 1060 to 1680 m. Precipitation averages ~490 mm annually, mostly occurring in winter and spring, with a pronounced summer drought. Average daily temperatures range from -3 ˚C in winter to 16 ˚C during the summer [10,17]. Seasonal cattle grazing has been the dominant land use on the ZPP for at least five decades [10]. At the turn of the 20th Century, the

entire PNB including the ZPP was homesteaded and used for cultivation and then largely abandoned and consolidated into larger parcels under private ownership [10]. Prescribed fire and targeted herbicide spraying for control of some non-native perennial forb species (e.g. *Hieracium caespitosum*, *Potentilla recta*) are other important land management practices.

Endress et al. (in Press [22]) used cluster analysis and non-metric multidimensional scaling to describe three plant communities in the ZPP as well as to relate patterns of native and non-native species abundance across those communities to each other and important abiotic/environmental correlates. This study used the same community classification developed by Endress et al. (in Press [22]): (1) the “xeric prairie” community consisted of the most water limited sites (highest heat load and greatest soil moisture deficits) and was dominated by *V. dubia*, *Poa secunda*, *Pseudoroegneria spicata* and *Danthonia unispicata*. This community aligned well with both the *Poa secunda*/*Danthonia unispicata* (i.e., shallow soil “scabflats”) and *Pseudoroegneria spicata/Poa secunda* plant association series described by Johnson and Simon (1987 [23]) and Johnson and Swanson (2005 [24]); (2) The “mesic prairie” community was dominated by *Festuca idahoensis*, *Koeleria macrantha*, *Pseudoroegneria spicata*, and numerous perennial forbs including *Geum triflorum* and *Arnica sororia*. This community also had the least non-native cover in 2008/2009, and aligned mostly with the *Festuca idahoensis*/*Koeleria macrantha* and *Festuca Idahoensis*/*Pseudoroegneria spicata* plant association series in the region [23-24]; and (3) the “old field” community consisted of flat or gently sloping sites with deep soils dominated by non-native perennial forage grasses including *Poa pratensis* and *Thinopyrum intermedium*. This community was strongly associated with past cultivation, i.e. old fields and did not align well with previous native plant community descriptions developed in the

region; however, the most abundant native species (*F. idahoensis*, *Pontentilla gracilis, Geum triflorum*) in the old fields were also among those dominant in the mesic prairie.

Non-native grass species are also among the most abundant and widespread species at the ZPP, but generally have not been used to define community variation, e.g., plant association classification (but see [20, 22]). In 2008/2009, non-native annual grasses (*V. dubia and Bromus spp.*) were strongly associated with the most water-limited sites. Both of these annual grasses were widespread across the xeric and mesic prairie; however *V. dubia* cover declined abruptly in the more mesic sites particularly within the old fields [22]. *Bromus* spp. were more uniformly distributed across the xeric and mesic prairie sites compared to *V. dubia*, but were similarly sparse in old fields [22]. Non-native perennial grasses including *Poa pratensis* and *Thinopyrum intermedium* dominated in relatively flat, mesic sites that were associated with old fields established long before the establishment of the preserve [22].

Field data

In order to evaluate vegetation change over the seven years, vascular plant composition of the most dominant species (based on foliar cover observed in 2008/2009; [22]) were re-sampled within 124 permanent plots (1 ha/each) across the prairie habitat (excluding canyonlands) of the ZPP. For the repeated sample, we focused on the most common species for our analyses (Table 1) in order to reduce observer error associated with measuring rare species, and to increase the number of plots that could be sampled. Further support for our decision to focus on the dominant species was provided by ordination analyses which showed that inclusion of more species did not substantially change ordination structure or findings from the initial (2008/2009) survey. Pilot sampling and review of TNC vegetation monitoring data [17]

occurred prior to the resampling effort to ensure that the 31 dominant species selected from the initial sampling effort were still the most abundant species in our study area and that other species had not increased enough to warrant inclusion. Any other vascular plant species encountered during sampling were recorded into functional groups (native perennial grass (NPG), native perennial forb (NPF), native annual grass (NAG), native annual forb (NAF), native sedge/rush, non-native perennial grass (NNPG), non-native perennial forb (NNPF), non-native annual grass (NNAG), non-native annual forb (NNAF)). Vegetation sampling took place during two different time periods, seven years apart: (1) summers of 2008 and 2009; and (2) summers of 2015 and 2016. Plot sampling within each time period was staggered one year so that the plots that were sampled in 2008 were resampled in 2015 and plots that were sampled in 2009 were measured in 2016 resulting in a seven year time gap between resamples for each plot. Within each plot, three line-point intercept transects (length = 50 m) were established in a spoke design, radiating out from the center of the plot at 0˚, 120˚, and 240˚ relative to magnetic North [22,25]. We observed species intercepts with transects at 1 m increments along transects for a total of 150 points sampled (50 per transect) per plot. Because multiple species are often encountered within different canopy layers at the same point, total cover can be > 100%. We calculated foliar cover for each species in a plot as the total number of hits for that species divided by 150 points multiplied by 100. Species frequency for a particular species was the total count of plots occupied by that species (maximum frequency possible = 124). Sampling occurred within three plant community types (xeric prairie, mesic prairie, old fields; [22]).

Data Analysis

Vegetation change over time

Paired t-tests were used to test the null hypothesis of no-change between non-native relative cover (proportional cover of non-native species relative to total plant cover expressed as a percentage) between sampling periods (plots were the sample units) for our entire study area and for each community type separately. Foliar cover change for functional groups (NPG, NPF, NNPG, NNAG) were calculated and compared to the null hypothesis of no change over time. Evidence of change in foliar cover was evaluated for all plots together and then separately for each community type. Bootstrapping was used (10,000 replicates) to generate 90 and 95% confidence intervals (basic bootstrap interval; [26]) for the mean change (mean of the paired differences; plots were sample units) in foliar cover between sample periods [27-28]. Strong evidence of change in foliar cover was indicated when 95% CIs did not cross zero; weak evidence for change was indicated when 90% CIs did not cross zero.

Blocked Multi Response Permutation Procedure (MRBP; [29-30]) was used to test the null hypothesis of no change in vascular plant species composition between time periods. MRBP is a nonparametric method for testing of no difference between two or more groups after accounting for block differences and provides an effect size chance-corrected within group agreement (*A*-statistic) that indicates within group homogeneity compared to random expectation [31]. An *A*-statistic value = 1.0 indicates that all components of a group are identical, while A=0 means within group homogeneity equals random expectation [31]. We used a Euclidean distance measure and aligned the medians to zero for all blocks (plots were the blocking factor). We performed separate tests for each community type.

Non-metric multidimensional (NMS; [30]) scaling using a Sorensen distance measure was used to extract the dominant community composition gradients from our dataset. We used the “slow and thorough” NMS autopilot setting and Kruskal’s strategy 1 for penalization of ties

in the distance matrix. A Sorensen distance measure was used to focus on relative differences in species abundances between sample units. Two dimensional ordinations were produced with a random starting configuration and a maximum of 500 iterations. We evaluated linear relationships between community composition variation and functional group abundance using a joint plot and identified community types on the ordination using convex hulls. The community types were defined by the 2008/2009 dataset (see [22] for methods). Temporal vectors that connect each plot between time periods were overlain onto the ordination and translated to the origin. This resulted in the tail of the vector (plot at the initial time of sampling) being located at the origin and the strength and direction of composition change for a plot being represented by the length and direction of the resulting vector respectively [31].

Species that changed the most in each community

Indicator species analysis (ISA) was performed, for each of the three community types separately to identify time period tendencies for species, i.e., to identify the species most responsible for driving compositional change between sampling periods [31-32]. ISA produces indicator values (IV) based on relative frequency (proportion of sample units from each group occupied) and abundance (proportional abundance for a particular species in a group relative to abundance in all groups) information that represent the strength of tendencies for species to occur within a priori groups [31]. Therefore, a species with a significant IV for time period one indicated higher abundance for that species during time period one (2008 & 2009) compared to time period two (2015 & 2016). Higher IV values indicate stronger affinity to a particular group (time period in this case), e.g., zero means a species is not an indicator; and 100 represents perfect indication, i.e., the species is always and only observed in that particular group [31]. Significance of IV's were determined by comparing observed values to results from 10,000

randomizations [30-31]. Changes in relative abundance and relative frequency over time were evaluated for identified indicator species. Additional 2-dimensional NMS ordinations were generated for the old field and mesic prairie communities (separately) to evaluate linear relationships between functional group change over time and community variation.

Ventenata dubia relationships with native/non-native plant abundance

Quantile regression was used to explore relationships between the foliar cover of the most abundant non-native species on ZPP, *V. dubia*, and the foliar cover of: (1) native perennials (forbs and grasses combined); (2) native perennial forbs; (3) native perennial grasses; (4) non-native perennial grasses; and (5) other non-native annual grasses separately [27,33]. Quantile regression is a method for estimating relationships between variables for all portions of the probability distribution, is non-parametric, and unlike ordinary least squares regression, does not require homogeneity of variance –an assumption not met in our dataset [34]. We evaluated relationships between the response and predictor variables for the 0.5 and 0.9 quantiles. This allowed us to evaluate functional relationships between variables at the median of the response distribution as well as near the maximum of the response distribution. We evaluated the 0.9 quantile because when there are many unmeasured factors that are potentially limiting, a common occurrence in natural systems, quantiles near the maximum response often provide more meaningful information regarding relationships between variables [34].

RESULTS

Vegetation change over time

Non-native relative cover increased across our study area from 27 to 30% over time (two sample paired t-test, two-tailed, $t = 2.6$, p-value = 0.01; Table 2). This change was primarily focused within the mesic prairie, where non-native relative cover increased from 16 to 22% (paired $t = 4.6$, $p < 0.001$; Table 2). There was weak evidence for a decrease (from 68 to 61%; paired $t = -1.5$, $p < 0.14$) in non-native relative cover in the old field community, and no evidence of change in the xeric prairie (paired $t = 0.45$, $p = 0.66$; Table 2). The two most dominant species in terms of cover (*F. idahoensis* and *P. spicata*) and frequency (*F. idahoensis* and *P. secunda*) were consistent over time. Other natives including *Lupinus* spp. (particularly in terms of frequency) and *Danthonia intermedia* (cover and frequency) showed decreased dominance over time (Table 1). Cover and frequency of *V. dubia*, increased substantially across our study area; *V. dubia* moved up a rank from fourth to third when evaluated based on cover (mean cover almost doubled over time from 7.6 to 14.9%, Table 1) and from eleventh to third for frequency, making it the third most abundant species in terms of cover and frequency in our study area (Table 1). Rank abundances for annual *Bromus* spp. and *P. pratensis* –the second and third most abundant non-natives- were relatively consistent over time. *T. intermedium* (frequency increased by 10%), and the annual forb *Veronica arvensis* (mean cover more than doubled from 0.8 to 1.8% and frequency increased by 27%) were two other non-native species that showed signs of increased dominance; however their mean cover and frequency were much lower than *V. dubia* (Table 1 and [35]).

Non-native annual grass cover increased from 13 to 20%, and native perennial forb cover decreased from 23 to 20% over time (Fig.1). There was weak evidence that native perennial grass cover increased from 55 to 58% (change = 3.1% 90% CI = 0.6, 6.0) since the initial sampling effort (Fig. 1). Increases in non-native annual grass mean cover appeared to be focused

primarily in the old field (increased from 4 to 16%) and mesic prairie communities (increased from 8 to 16%; Fig 1). Mean native perennial forb cover decreased (from 30 to 25%) in the mesic prairie (Fig. 1). Mean native perennial grass cover increased substantially (from 12 to 27%) in old fields (Fig. 1), and was consistent with an increase in total vascular plant cover (11.6%, 95% CI = 2.4, 20.7). There was no evidence for change in cover for any functional group within the xeric prairie (Fig 1). We also calculated the total change in native perennial forb cover (not limited to the dominant species) to make sure that trends in dominant forbs tracked the entire community. Consistent with the dominant forbs, all native perennial forbs decreased by 5% (95% CI = -7.6, -2.9) in our sample area, and the greatest decrease for all native perennial forb foliar cover (-6.6, 95% CI = -9.9, -3.2) was observed in the mesic prairie.

Vegetation composition changed over time in all three plant communities ($p < 0.001$; Table 3). The greatest changes were detected in old fields ($A = 0.07$) followed by the mesic prairie ($A = 0.05$), and lastly by the xeric prairie ($A = 0.03$; Table 3). Temporal vectors extracted from the NMS ordination indicated that most old field plots shifted to lower Axis 1 (explaining 64% of variation in the distance matrix) scores over time which was associated with lower composition of non-native perennial grass species (Fig. 2). The mesic prairie plots tended to shift down along Axis 2 (explaining 23% of variation in the distance matrix), consistent with increasing non-native relative abundance, and no clear direction of change was observed for the temporal vectors generated from xeric prairie plots (Fig. 2).

Species that changed the most in each community

Ventenata dubia was among the strongest indicator species for the most recent sampling effort in all community types, and the only non-native that increased abundance across our entire

study area, particularly within the mesic prairie and old field communities (Table 4). Dominant species compositional changes in the old fields included decreased abundances of two non-native perennial grasses (*Poa compressa* and *P. pratensis*) and native perennial forb species (*Achillea millifolium, Lupinus* spp.) and increased abundances of *F. idahoensis, V. dubia* (rank abundance in old fields changed from 19th to 4th over time; [35]), and *Geum triflorum* (Table 4*).* Decreases in bare ground cover were correlated with increases in cover of *F. idahoensis* (r = -0.12), *V. dubia* (r = -0.41) and *G. triflorum* (r = -0.32). Within the old field community, *V. dubia*, native perennial grass, and native perennial forb increases tended to occur in the same area of ordination space, and were negatively associated with non-native perennial grass cover in old fields (Fig 3);

Dominant species compositional changes in the mesic prairie included substantial increases in non-native species abundance (*V. dubia*, *V. arvensis, T. intermedium)* and decreased native perennial forb (*Lupinus spp*., *A. sororia, P. gracilis, Hieracium cynoglossoides, Geranium viscosissimum)* cover and potentially subtle declines in native bunchgrass (*F. idahoensis*, *P. spicata*) abundance (Table 4). *Ventenata dubia* dominance increased substantially (rank abundance in the mesic prairie changed from 11th to 3rd over time; [35]). Native perennial forbs showed a slight tendency to decrease in plots with higher initial non-native annual grass cover as well as where non-native annual grass cover increased over time (Fig 3).

Notable changes in species abundance within the xeric prairie included increased abundances of two non-native grass species (*V. dubia*, *P. compressa*). Other species that showed significant changes in abundance in the xeric prairie were those that tended to be associated with the more mesic communities and were sparse in the xeric prairie community [35], i.e., increased abundance of one native perennial grass (*Bromus carinatus*), and decreased abundances of native

perennial forb (*A. sororia*, *Phlox spp*., *P. gracilis*) and grass species (*K. macrantha*, *D. intermedium;* Table 4).

Relationship between *V. dubia* and native perennial cover

Ventenata dubia cover was negatively related to native perennial cover and native perennial forb cover for both sampling periods (Fig. 4 & Table 5). The greatest negative slopes were detected for the 0.9 quantile followed by the 0.5 quantile (Fig 4 & Table 5). The slopes for responses in both quantiles decreased over time particularly in the 0.9 quantile (Fig. 4 & Table 5). Relationships between *V. dubia* cover and native perennial grass cover were inconsistent between years and across quantiles. *V. dubia* cover was negatively related to native perennial grass cover for the 0.9 quantile during the first sampling period but not the 0.5 quantile and for the 0.5 quantile during the second sampling period, but not the 0.9 quantile (Table 5). Because vegetation trends in the old field community were much different than those in the uncultivated prairie, analyses were also performed for the uncultivated prairie alone; however, this did not change our findings or interpretation of the data. Inconsistent relationships between *V. dubia* and native perennial grasses over time and quantiles suggest complex interactions between these two variables and may be related to factors such as lag time effects between species interactions, variation in native species responses, differential responses by community type, or may reflect a weak relationship between these variables that cannot be distinguished from noise in the data. *V. dubia* was negatively related to non-native perennial grass cover during both sampling periods. The greatest negative slope (coefficient = -1.3, 95% CI = -1.4, -1.1) was observed for the 0.9 quantile during the first sampling period. Slopes for both quantiles decreased substantially and were similar for the second sampling period where it was estimated that a one percent increase in *V. dubia* cover corresponded to ~ 0.1% decrease in non-native perennial grass cover for the 0.5

and 0.9 quantiles (Table 5). *V. dubia* was positively related to cover of other non-native annual grasses for the 0.5 quantiles during both sampling periods. It was estimated that a 1% increase in *V. dubia* cover corresponded to approximately a 0.1% increase in the median cover of other non-native annual grasses (Table 5).

Discussion

Our study revealed three major findings. First, we found evidence of increased relative cover (~ 7%) of native vegetation in old fields, mostly due to increased *F. idahoensis* (increased from an average of 5 to 16%) and *G. triflorum* abundance (increased from 3 to 7%; [34]). Our observations were contrary to a previous study, where native perennial bunchgrass cover did not change consistently over time in ZPP's old fields [17], but is consistent with Taylor and Schmalz's (2012; [17]) hypothesis that *F. idahoensis* abundance would continue to increase in old fields over time. Despite increasing cover of some native species in old fields, two lines of evidence suggest that a continued shift back to native species dominance may not occur: 1) we found no evidence that non-native perennial grass cover has changed over time. In one way this is promising for conservation efforts because it suggests that the non-native perennial grasses have not expanded their distributions. Yet, this finding also suggests that they have not given ground to colonizing vegetation and aligns with previous observations which indicate that non-native perennial forage grasses seeded after disturbance can be highly competitive with colonizing native species and persist on the landscape [36-40]; and 2) *V. dubia* has spread into and quickly become a dominant species in ZPP's old fields [35]. Cultivation of dry land sites, similar to the ZPP, can leave long lasting impacts on site characteristics [17,37,41-42], that can facilitate invasion by non-native species [43] and in some cases may result in the crossing of

thresholds which make recovery to pre-disturbance communities unlikely [37,42,44-45]. Cultivation legacies (e.g., increased cover of bare-ground) may have facilitated the expansion of *V. dubia* into old fields. For example, change in *V. dubia* foliar cover was negatively related (r = -0.41) to change in cover of bare ground in our study. Old fields in the PNB canyon grasslands contain a higher dominance of *V. dubia* in the soil seed bank than similar adjacent noncultivated sites, suggesting that, once introduced, this species is well positioned for continued dominance when native seed is limited [21]. However, our observations that *V. dubia* has continued to spread into as well as become a dominant species in the previously uncultivated mesic prairie that was dominated by native perennial vegetation during our initial sampling effort (2008/2009) suggests that *V. dubia* invasion into the most mesic grassland sites in the ZPP is not dependent on cultivation related disturbance and will likely remain a dominant component in ZPP's old fields –given current conditions, management, and land-use practices- even if native vegetation continues to recolonize that community.

Given our results, we suspect that restoration efforts in ZPP's old fields that reduce non-native perennial grass cover and open up bare ground have the potential for re-colonization by both native vegetation (i.e. *F. idahoensis* and *G. triflorum*) and/or *V. dubia*, and will probably result in colonization by both. Little is known about *V. dubia* impacts on native vegetation abundance and composition or the thresholds/potential to shift dominance from one functional group (native perennial, non-native perennial, or non-native annual) to another in PNB grasslands. We recommend that future research include the identification of potential community states and thresholds for transitions between different communities in PNB grasslands. Within old fields in particular, analyses comparing outcomes for different potential communities may be helpful when considering restoration/management options. For example,

these types of analyses may help practitioners address questions such as: would conservation of non-native perennial grass dominated communities limit the spread of *V. dubia*, and provide more desired ecosystem functionality compared to other possible states, in old fields?; or, are other potential communities such as a "native perennial grass/*V.dubia*" co-dominant community possible, and if so, would such a community be more beneficial for conservation of biodiversity?

Our second important finding is that within previously uncultivated mesic prairie, that non-native abundance –primarily *V. dubia* – increased (relative cover increased by ~ 6%; foliar cover increased by a factor of 4) at the same time that native perennial forb abundance declined (from 30 to 25%). Regardless of whether or not these two responses have a causal relationship, both responses –if they are indicative of longer-term trends– may have important consequences to the ecological integrity of the ecosystem. Increased dominance of non-native annual grasses is likely to have substantial impacts on community level functionality including composition, diversity, and soil characteristics of the invaded system [6]. Conversions of bunchgrass dominated communities to non-native annual grasslands in other western North American ecosystems have been associated with reduced floral and faunal diversity, decreased forage production for grazing animals, reduced soil stability, increased susceptibility to erosion, and alteration to disturbance regimes (e.g., increased fire frequency and intensity), nutrient cycling dynamics, and species/structural composition [5,46-51]. Most examples of non-native annual grass impacts on bunchgrass communities in the Intermountain West have focused on annual *Bromus* species (e.g. *Bromus tectorum*; [48,52]). To date, few studies have investigated invasion dynamics and impacts of *V. dubia* [53]. Those that have, suggest potential competitive displacement of native vegetation by *V. dubia* [54, 55], reduced forage production, evidence of a negative association between *V. dubia* and biodiversity in a northern Idaho grassland [53], and a

potentially broad ecological niche for *V. dubia* within bunchgrass, shrubland, and open forest communities in the Pacific Northwest [24,53,55-56]. Within Sagebrush-steppe habitats, *V. dubia* cover has been positively associated with mesic upland sites [55] and negatively associated with bio-crust cover and lichen richness [57]. Thus far, research has not identified linkages between fire and *V. dubia* invasion in the PNB [58]. Further research is needed to inform land managers about the potential impacts of *V. dubia* on invaded PNB communities, and whether or not such impacts will mirror those of other annual grass invasions in western North America.

Our detection of a small decline in native perennial forb cover may be an early indication of a longer term trend in native vegetation change; however, we suggest caution in interpretation of the ecological significance of this result for a couple of reasons. First, our study is based on change between two time periods, and a 5% change in foliar cover is a relatively small change that may be attributable to some unmeasured natural variation in the system; future research will be needed to determine long-term trajectories of perennial forb abundance as well as species specific responses. Second, we do not expect that an overall ~5% decline in perennial forb cover will have significant impacts to ecosystem function or services in the ZPP. We recommend that trends in native perennial forb abundance be closely monitored at the ZPP because continued declines of forb cover may have negative cascading effects in the ZPP. Native perennial forbs serve important functions in PNB grasslands. Forbs are extremely important for floral diversity in PNB prairies because they are the major source of floral richness (make up > 60% of vascular plant species in the ZPP) in these and similar grasslands [14, 59-60]. Changes in native perennial forb abundance can impact species diversity and abundance at higher tropic levels, i.e., arthropod diversity and grassland birds [61-63]. Pollinator populations are particularly sensitive to changes in forb composition and abundance because forbs increase habitat and forage

diversity for pollinators, and their flowers are the primary food source for adult stage pollinators in grasslands [15, 64]. Forbs have novel functional traits in PNB grassland systems including nitrogen fixation by legumes (e.g. *Lupinus* spp) and the presence of taproots which differ in their interaction with soil properties (e.g. water infiltration) compared to fibrous roots of grasses. Reductions in such functional traits can influence above and below ground floral and faunal composition and diversity, habitat, soil physical characteristics, and nutrient dynamics [65-66]. Additionally, many PNB forbs are culturally important resources (e.g. foods and medicines) for Native American tribes in the region [67-68].

Thirdly, *V. dubia* has invaded, and become a dominant species in all three communities (rank abundance = 1, 3, & 4 for the xeric, mesic, and old field communities respectively [35]), and its abundance was negatively related to native perennial forb abundance. Our finding that *V. dubia* has rapidly spread into the mesic prairie and become a dominant species - 3rd in rank abundance and 6th in rank frequency - absent fire or major anthropogenic disturbances (heavy grazing pressure or known cultivation) suggest that previous hypotheses of high resistance of the more mesic PNB communities to invasion by non-native annual *Bromus* spp. may not apply to *V. dubia* [23,24]. Because this was an observational study, we do not know whether or not *V. dubia* invasion is responsible for the decline in native perennial forbs that we observed. Previous evidence that annual grasses have reduced native and non-native perennial forb abundance through competition for light in similar grasslands [69] coupled with our detection of a negative association between *V. dubia* and native perennial forb cover suggest that a causal link between increased *V. dubia* cover and decreased native forb cover should be explored further. Of course, both of these responses may be consequences of some other factor that is driving change in our study area. Other important disturbances to the ZPP system including livestock grazing (a novel

disturbance compared to evolutionary pressures), wild ungulate herbivory, and fire (a regime greatly altered by human activity) can have profound impacts on vegetation composition and may play important roles in vegetation change that we detected. Additionally, long-term changes in climate may influence changing distributions of species in our study area [70-71]. Future experimental studies that consider the impact of livestock grazing, wild ungulate herbivory, fire, and climate will aid the identification of drivers for *V. dubia* spread as well as the potential for a causal linkage between *V. dubia* invasion and native vegetation change.

Conclusion

This effort is one of few peer-reviewed studies to evaluate changes in native and non-native plant species composition over time in a PNB grassland (but see [20,58]). Our results demonstrate that plant composition of ZPP's old fields are shifting towards that of the uncultivated grassland plots. Such change in old fields, however, may not represent progress towards native dominated communities because our findings suggest that non-native perennial grass cover has not changed and previous bare ground has not only been colonized by native species but also by *V. dubia* which is now the fourth most dominant species in old fields. Future research is needed to better understand the potential plant community states in PNB old fields, how different community states may aid/hinder management objectives (e.g., conservation of biodiversity), as well as management practices that influence transitions between potential community states. While we found varying responses of native vegetation across community types, in general, our findings indicate that despite the protected status of the ZPP, that *V. dubia* (an annual non-native grass) has rapidly spread into and become a dominant species within all major grassland community types (xeric, mesic, and old fields) in the ZPP. Previous research in

similar grasslands have consistently demonstrated that shifts from native bunchgrass to non-native annual grass dominance greatly alter ecosystem functionality and are generally associated with suppressed native species richness and abundance. Such knowledge combined with our findings that *V. dubia* cover was negatively related to native perennial forb cover, as well as our detection of potential declines in native perennial forb abundance in the most recently invaded grassland sites suggest that *V. dubia* poses a threat to conservation of biodiversity and grassland function in the largest remaining protected Pacific Northwest Bunchgrass remnant.

Acknowledgements

We thank Kent Coe, Andie Lueders, Tyler Dungannon, Kolby Haliewicz, Walker Powell, Joanna Patten, Luke Ridder, Brogan Watson, Ashtin Olsen, Ngeyaol Polycarp, and Shanda Zettles for assistance collecting field data, and The Nature Conservancy for their collaboration and support for this project. Funding was provided by USDA NRI Competitive Grant Agreement No. 2006-35320-17244 (BE), USDA Forest Service Pacific Northwest Research Station # 05-JV-11261967-069 (BE), the Oregon State University Agricultural Research Foundation (LM, BE), and the Oregon State University Agriculture Experiment Station (LM, BE).

References

1. Henwood WD. 2010. Toward a strategy for the conservation and protection of the world's temperate grasslands. Great Plains Research. 2010; 20:121–134.
2. Samson, F, Knopf F. Prairie conservation in North America. BioScience. 1994; 44:418–421.

3. Hoekstra JM, TM Boucher, Ricketts TH, Roberts C. Confronting a biome crisis: global disparities of habitat loss and protection: Confronting a biome crisis. Ecology Letters. 2004; 8:23–29.
4. Foxcroft LC, Pysek P, Richardson DM, Pergl J, Hulme PE. The bottom line: impacts of alien plant invasions in protected areas. eds, Plan invasions in protected areas: patterns, problems and challenges, Invading Nature. Springer Series in Invasion Ecology. 2013; 7, DOI 10.1007/978-94-007-7750-7_2.
5. D'Antonio CM, Vitousek PM. Biological invasions by exotic grasses, the grass/fire cycle, and global change. Annual Review of Ecology and Systematics. 1992; 23:63-87.
6. Pyšek P, Jaroski V, Hulme PE, Pergl J, Hejda M, Schaffner U, Vila M. A global assessment of invasive plant impacts on resident species, communities and ecosystems: the interaction of impact measures, invading species' traits and environment. Global Change Biology. 2012; 18:1725-1737.
7. Rejmanek, M., Richardson, DM, Pyšek P. Plant invasions and invasibility of plant communities. Vegetation Ecology. 2013; Second Edition. John Wiley and Sons, LTD: 387-424.
8. Gilbert, B, Levine M. Plant invasions and extinction debts. Proceedings of the National Academy of Sciences. 2013; 110:1744-1749.
9. Tisdale EW. Grasslands of Western North America: the Pacific Northwest Bunchgrass. Pages 232-245 in A. C. Nicholson, A. McLean, and T. E. Baker, editors. Proceedings of the 1982 grassland ecology and classification conference. British Columbia Ministry of Forests, Kamloops, B. C. 1982.

10. Bartuszevige AM, Kennedy PL, Taylor RV. Sixty-seven years of landscape change in the last, large remnant of the Pacific Northwest Bunchgrass Prairie. Natural Areas Journal. 2012; 32:166-170.

11. Dixon AP, Faber AP, Langendoen D, Josse C, Morrison, J, Loucks, CJ. Distribution mapping of world grassland types. Journal of Biogeography. 2014; 41:2003–2019.

12. Noss RF, LaRoe ET, Scott JM. Endangered ecosystems of the United States: a preliminary assessment of loss and degradation. National Biological Service Biological Report 28. 1995. US Department of the Interior, Washington, D.C., U S A.

13. Tubbesing C, Strohm C, DeBano SJ, Gonzalez N, Kimoto C, Taylor RV. Insect visitors and pollination ecology of Spalding's Catchfly (*Silene spaldingii*) in the Zumwalt Prairie of Northeastern Oregon. Natural Areas Journal. 2014; 34:200-211.

14. Darambazar E, DelCurto T, Damiran D, Clark AA, Taylor RV. Species composition and diversity on northwestern bunchgrass prairie rangelands. Proceedings of Western Section, American Society of Animal Sciences. 2007; 58:233-236.

15. Kimoto C, DeBano SJ, Thorp RW, Taylor RV, Schmalz H, DelCurto T, Johnson T, Kennedy PL, Rao S. Short-term responses of native bees to livestock and implications for managing ecosystems services in grasslands. Ecosphere. 2012; 3:88.

16. Johnson, TN, Kennedy PL, DelCurto T, Taylor RV. Bird community responses to cattle stocking rates in a Pacific Northwest bunchgrass prairie. Agriculture, Ecosystems, and Environment. 2011; 144:338-346.

17. Taylor RV, Schmalz HJ. Monitoring of upland prairie vegetation on the Zumwalt Prairie Preserve 2003-2011. The Nature Conservancy Northeast Oregon Field Office, Enterprise, OR. 2012.

18. Taylor RV, Pokorny ML, Mangold J, Rudd N. Can a combination of grazing, herbicides, and seeding facilitate succession in old fields? Ecological Restoration. 2013; 31:141-143.

19. Kennedy PL, DeBano SJ, Bartuszevige AM, Lueders AS. Effects of native and non-native grassland plant communities on breeding passerine birds: implications for restoration of northwest bunchgrass prairie. Restoration Ecology. 2009; 17:515-525.

20. Bernards SJ, Morris LR. Influence of topography on long-term successional trajectories in canyon grasslands. Applied Vegetation Science. 2017; 20:236-246.

21. Bernards SJ, Morris LR. Comparisons of canyon grassland vegetation and seed banks along an early successional gradient. Northwest Science. 2017; 91:27-40.

22. Endress BA, Averett JP, Naylor BN, Morris LR, Taylor RV. Non-native species threaten the biotic integrity of the largest remnant Pacific Northwest Bunchgrass Prairie in the United States. In Press. Applied Vegetation Science.

23. Johnson CG, Simon SA. Plant associations of the Wallowa-Snake province. US Dept. of Agriculture, Forest Service, Pacific Northwest Region. 1987.

24. Johnson CG, Swanson DK. Bunchgrass plant communities of the Blue and Ochoco Mountains: a guide for managers. US Department of Agriculture, Forest Service. General Technical Report, PNW-GTR-641. 2005.

25. Herrick JE,Van Zee J, Havstad K, Burkett L,Whitford WN. Monitoring manual for grassland, shrubland and savanna ecosystems, Volume I: quick start. : Tucson, Arizona: The University of Arizona Press. 2005.

26. Davison AC, Hinkley DV. Bootstrap methods and their application, Chapter 5. Cambridge University Press. 1997.

27. R Core Team. R: a language and environment for statistical computing. R Foundation for Statistical Computing, Vienna. <http://www.R-project.org>. 2015.

28. Canty A, Ripley B. Boot: Bootstrap R (S-Plus) Functions. R package version 1.3-18. 2016.

29. Mielke PW Jr. Meteorological applications of permutation techniques based on distance functions. In P. R. Krishnaiah and P. K. Sen, eds., Handbook of Statistics. 1984; 4:813-830. Elsevier Science Publishers.

30. McCune B, Mefford MJ. PC-ORD. Multivariate analysis of ecological data version 7.0. MjM Software, Gleneden Beach, Oregon, U S A. 2015.

31. McCune B, Grace JB. Analysis of ecological communities. MJM Software, Gleneden Beach, Oregon. USA. 2002.

32. Dufrene M, Legendre MP. Species assemblages and indicator species: the need for a flexible asymmetrical approach. Ecological Monographs. 1989; 67:345–366.

33. Koenker R. Quantreg: Quantile Regression. R package version 5.35. https://CRAN.R-project.org/package=quantreg. 2018.

34. Cade BS, Noon BR. A gentle introduction to quantile regression for ecologists. Frontiers in Ecology and the Environment. 2003; 1:412-420.

35. Averett JP, Morris LR, Endress BA. Dataset of plant composition change over seven years at the Zumwalt Prairie Preserve, Oregon, USA. Data In Brief. In Review.

36. Keeley JE. Fire management impacts on invasive plants in the western United States. Conservation Biology. 2006; 20(2):375-384.

37. Kulmatiski A. Exotic plants establish persistent communities. Plant Ecology. 2006; 187:261-275.

38. Pickering C, Hill W. Roadside weeds of the Snowy Mountains, Australia. Mountain Research and Development. 2007; 27(4):359-367.

39. McDougall KL, Khuroo AA, Loope LL, Parks CG, Pauchard A, Reshi ZA, Rushworth I, Kueffer C. Plant invasions in mountains: global lessons for better management. Mountain Research and Development. 2011; 31(4):380-387.

40. Morris LR, Monaco TA, Sheley RL. Impact of cultivation legacies on rehabilitation seedings and native species re-establishment in Great Basin desert shrublands. Rangeland Ecology and Management. 2014; 67:285-291.

41. Morris LR, Monaco TA, Sheley RL. Land-use legacies and vegetation recovery 90 years after cultivation in Great Basin sagebrush ecosystems. Rangeland Ecology and Management. 2011; 64:488-497.

42. Morris, LR, Monaco TA, Blank R, Sheley RL. Cultivation legacies in soils after rehabilitation seeding in the Great Basin, USA. Arid Land Research and Management. 2016; 30:362-374.

43. Endress BA, Naylor BJ, Parks CG, Radosevich SR. Landscape factors influencing the abundance and dominance of the invasive plant Potentilla recta. Rangeland Ecology and Management. 2007; 60:218-224.

44. Morris LR, Monaco TA, Blank R, Sheley RL. Long-term redevelopment of resource islands in shrublands of the Great Basin, USA. Ecosphere. 2013; 4:1-14.

45. Cramer VA, Hobbs RJ. Standish RJ. What's new about old fields? Land abandonment and ecosystem assembly. Trends in Ecology and Evolution. 2008; 23:104-112.

46. Seabloom EW, Horpole WS, Reichman OJ, Tilman D. Invasion, competitive dominance, and resource use by exotic and native California grassland species. Proceedings of the National Academy of Sciences. 2003; 100:13384-13389.

47. Mack RN. Invasion of *Bromus tectorum* L. into western North America: an ecological chronicle. Agro-ecosystems. 1981; *7*:145-165.

48. Mack RN. Temperate grasslands vulnerable to plant invasions: characteristics and consequences. Biological Invasions a Global Perspective. Edited by J. A. Drake et al. Published by John Wiley & Sons Ltd. 1989.

49. Davies KW. Plant community diversity and native plant abundance decline with increasing abundance of an exotic annual grass. Oecologia. 2011; 167:481-491.

50. Balch JK, Bradley BA, D'Antonio CM, Gomez-Dans J. Introduced annual grass increases regional fire activity across the arid western USA (1980-2009). Global Change Biology. 2013; 19:173:183.

51. Freeman ED, Sharp TR, Larsen RT, Knight RN, Slater SJ, McMillan BR. Negative effects of an exotic grass invasion on small-mammal communities. PLoS ONE. 2014; 9:e108843.

52. Brooks ML, Brown CS, Chambers JC, D'Antonio CM, Keeley JE, Belnap J. Exotic annual *Bromus* invasions: comparisons among species and ecoregions in the western United States. MJ Germino et al. (eds.) Exotic Brome-Grasses in Arid and Semiarid ecosystems of the western US, Springer series on Environmental Management. 2016.

53. Wallace JM, Pavek PL, Prather TS. Ecological characteristics of *Ventenata dubia* in the Intermountain Pacific Northwest. Invasive Plant Science and Management. 2015; 8:57-71.

54. McKay S, Morris LR, Morris C, Leger E. Examining the competitive effects of Ventenata dubia. Prairie Naturalist. 2017; 49:19-22.

55. Jones LC, Norton N, Prather TS. Indicators of Ventenata (*Ventenata dubia*) invasion in sagebrush steppe rangelands. Invasive Plant Science and Management. 2018; 11:1-9.

56. Averett JP, McCune B, Parks CG, Naylor BJ, DelCurto T, Mata-González R. Non-native plant invasion along elevation and canopy closure gradients in a Middle Rocky Mountain ecosystem. PLoS ONE. 2016; 11(1): e0147826: https//doi.org/10.1371/journal.pone0147826.

57. Cellini, J. Variation in the relationship between invasive annual grasses and biological soil crust across a rainfall gradient. Thesis. Eastern Washington University. 2016.

58. Ridder LW. The response of *Ventenata dubia* to prescribed fire and ungulate grazing on the Pacific Northwest Bunchgrass Prairie. Thesis. Oregon State University, Corvallis, OR. 2019.

59. Pokorny ML, Sheley RL, Svejcar TJ, Engel RE. Plant species diversity in a grassland plant community: evidence for forbs as a critical management consideration. Western North American Naturalist. 2004; 64:219-230.

60. Daubenmire RF, An ecological study of the vegetation of southeastern Washington and adjacent Idaho. Ecological Monographs. 1942; 12:53-79.

61. Dion N, Hobson KA, Larivière. Interactive effects of vegetation and predators on the success of natural and simulated nests of grassland songbirds. The Condor. 2000; 3:629-634.

62. Haddad NM, Crutsinger GM, Gross K, Haarstad J, Knops JMH, Tilman D. Plant species loss decreases arthropod diversity and shifts trophic structure. Ecology Letters. 2009; 12:1029-1039.

63. Andersen EM, Cambrelin MN, Steidl RJ. Responses of grassland arthropods to an invasion by nonnative grasses. Biological Invasions. 2018. https://doi.org/10.1007/s10530-018-1831-z

64. Potts SG, Woodcock BA, Roberts SPM, Tscheulin T, Pilgrim ES, Brown VK, Tallowin JR. Enhancing pollinator biodiversity in intensive grasslands. Journal of Applied Ecology. 2009; 46:369-379.

65. Tilman D, Reich PB, Knops J, Wedin D, Mielke T, Lehman C. Diversity and productivity in a long-term grassland experiment. Science. 2001; 294:843-845.

66. Gould IJ, Quinton JN, Weigelt A, De Deyn GB, Bardgett RD. Plant diversity and root traits benefit physical properties key to soil function in grasslands. Ecology Letters. 2016; 19:1140-1149.

67. Moreman DE. Native American Ethnobotany. Portland, OR. Timber Press Inc. 927 pages. 1998.

68. Hunn ES, Morning Owl T, Cash Cash PE, Engum JK. Cáw Pawá Láakni: They are Not Forgotten. Tamástslikt Cultural Institute. Pendleton, Oregon. 2015.

69. Coleman HM, Levine, JM. Mechanisms underlying the impacts of exotic annual grasses in a coastal California meadow. Biological Invasions. 2007; 9: 65-71.

70. Bradley BA, Blumenthal DM, Wilcove DS, Ziska LH. Predicting plant invasions in an era of global change. Trends in Ecology and Evolution. 2010; 25:310-318.

71. Bansal S, James JJ, Sheley RL. The effects of precipitation and soil type on three invasive annual grasses in the western United States. Journal of Arid Environments. 2014; 104:38-42.

Figures

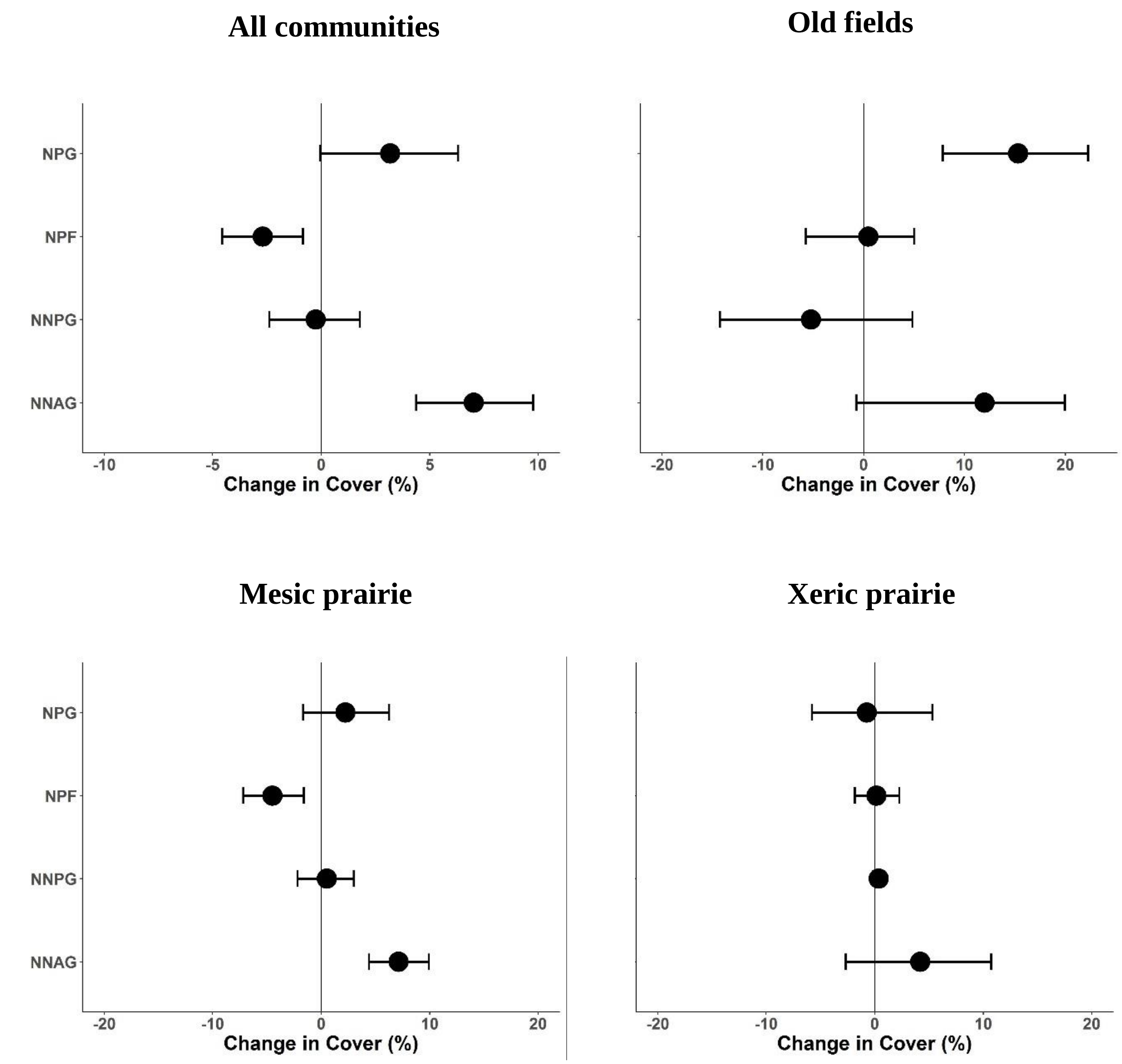

Figure 1: Foliar cover change (paired differences) between 2008/2009 and 2015/2016 for dominant vegetation functional groups (NPG, native perennial grass; NPF, native perennial forb; NNPG, non-native perennial grass; NNAG, non-native annual grasses) in the Zumwalt Prairie Preserve. Points are means, error bars contain 95% confidence intervals (bootstrap, 10,000 replicates, percentile method) around the paired difference in the means between the two time periods, and vertical lines indicate zero change.

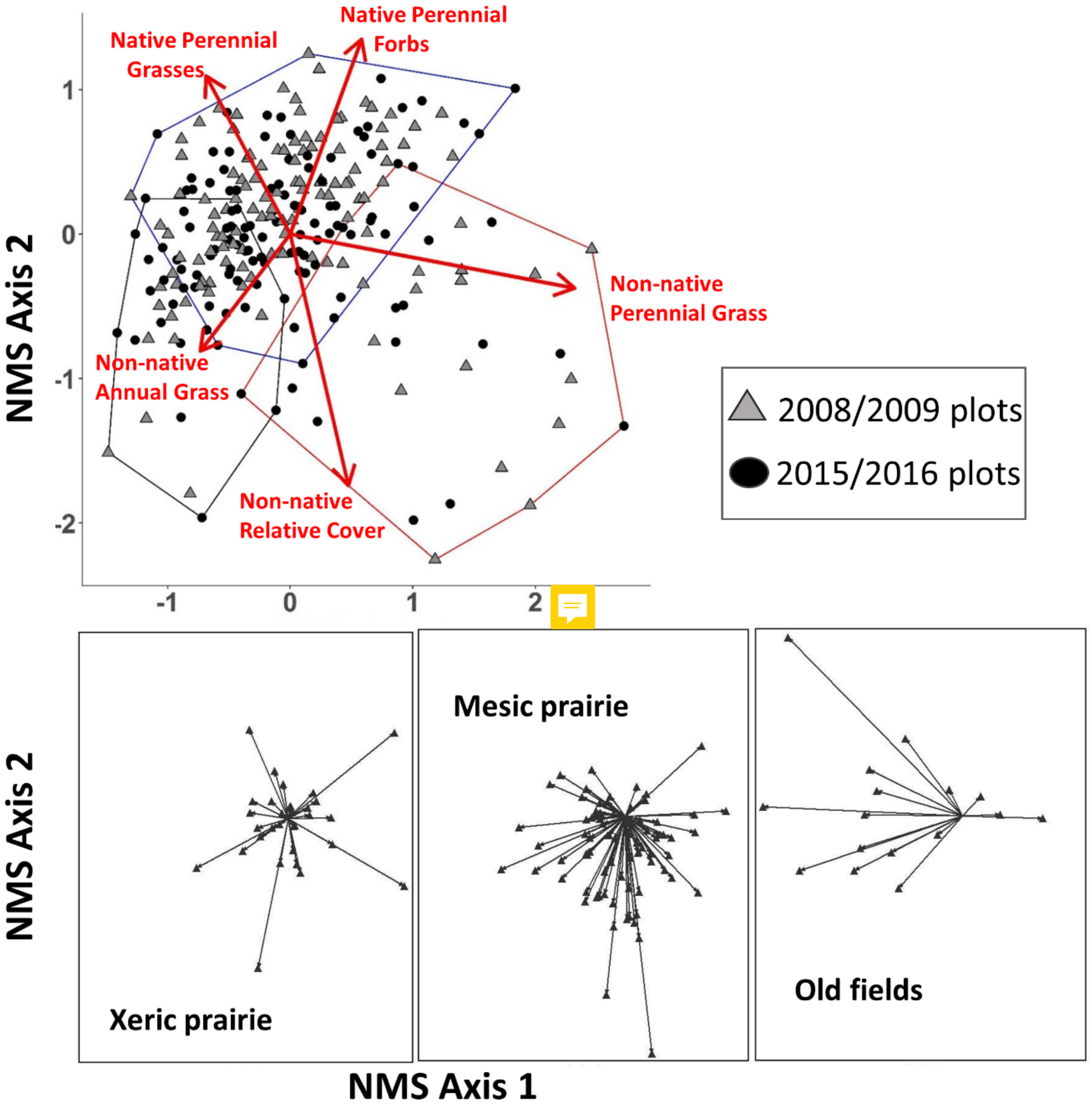

Figure 2: NMS ordination of plots in species space. (Top panel) Joint plot with vectors showing direction and magnitude (length) of linear correlations between functional group abundance (% cover) and the ordination space. Convex hulls show the three community types (red, old field community; blue, mesic prairie community; black, xeric prairie community). (Bottom panels) Temporal vectors showing direction and magnitude of change in ordination space for each plot within three grassland communities. Axis one and two explained 64 and 23% of variation in the distance matrix respectively.

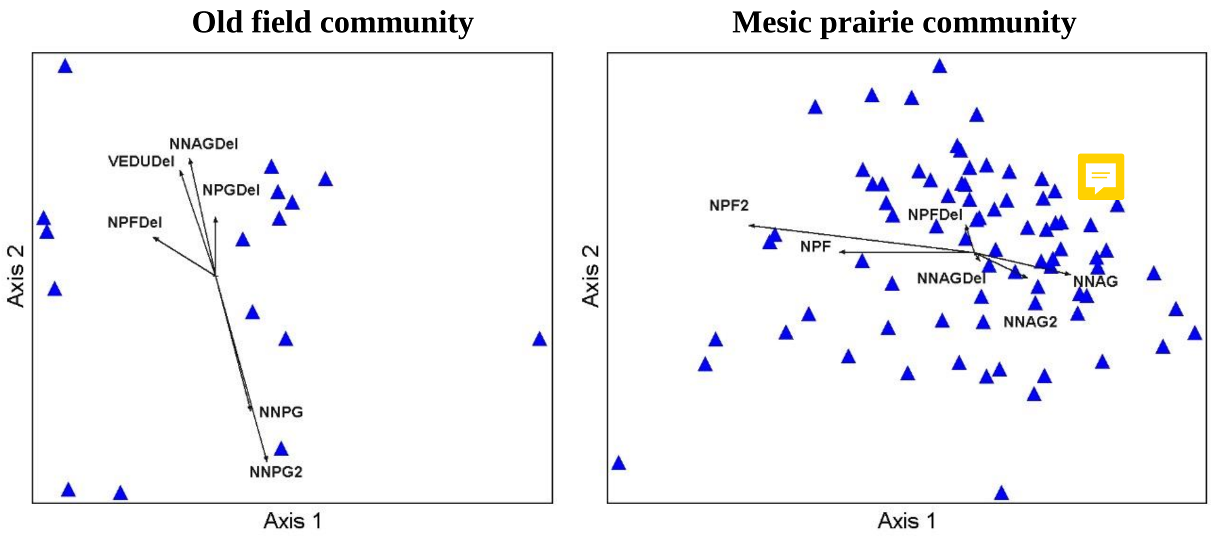

Figure 3: NMS ordinations of the old field and mesic prairie communities using abundance data from 2015/2016. The direction and length of the vectors indicate direction and strength of linear correlations between variables and the ordination space. NPG (native perennial grass); NNPG (non-native perennial grass); NPF (native perennial forb); NNAG (non-native annual grass); VEDU (*Ventenata dubia*). “Del” signifies difference between time periods, and a “2” indicates the second time period, e.g., NNPG is the cover of non-native perennial grasses for the 1st time period (2008/2009) and NNPG 2 is cover for non-native perennial grasses for the 2nd time period (2015/2016)

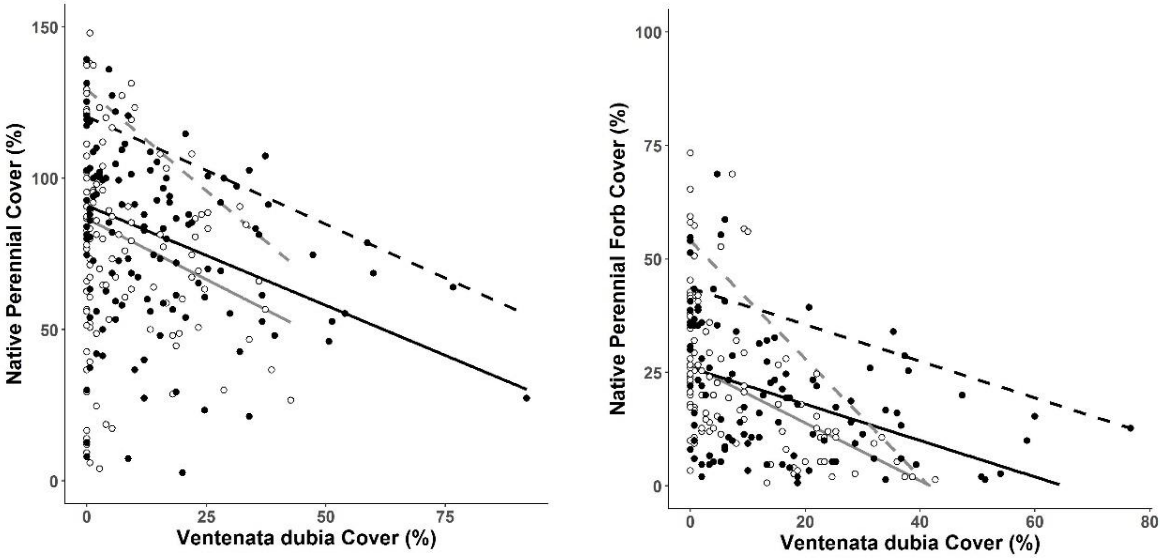

Figure 4: Scatter plots of *Ventenata dubia* foliar cover against native perennial grass and forb foliar cover (left panel) and native perennial forb foliar cover (right panel). White points and grey lines indicate data and regression fits from the 2008/2009 sampling period, and black points and black lines show data from the 2015/2016 sampling period. Solid lines are regression lines for the 0.5 quantile and dashed lines are regression lines for the 0.9 quantile.

TABLES

Table 1: Species abundance summaries for dominant vascular plants occurring in the Zumwalt Prairie Preserve study area ordered top to bottom by rank abundance (% cover) from initial time period (T1, 2008/2009). Log abundance (Log Abund) was calculated as the log (base 10) of the total summed abundance for a particular species in all plots. T2 is the second time period (2015/2016). Non-native species are listed in bold text.

	Rank Abund		Log Abund		Rank Freq		Freq		Mean Cover (%)	
Species	T1	T2	T1	T2	T1	T2	T1	T2	T1	T2
Festuca idahoensis	1	1	3.47	3.49	1	1	122	123	24.0	24.8
Psuedoroegneria spicata	2	2	3.30	3.30	4	4	107	107	16.3	16.1
Poa secunda	3	4	3.00	3.05	2	2	116	112	8.1	9.1
Ventenata dubia	**4**	**3**	**2.97**	**3.27**	**11**	**3**	**84**	**108**	**7.6**	**14.9**
Poa pratensis	**5**	**6**	**2.87**	**2.85**	**12**	**13**	**75**	**72**	**6.0**	**5.8**
Geum triflorum	6	5	2.86	2.95	10	9	87	91	5.9	7.2
Bromus spp.	**7**	**7**	**2.79**	**2.67**	**8**	**8**	**95**	**93**	**5.0**	**3.8**
Arnica sororia	8	9	2.71	2.58	5	7	104	93	4.1	3.1
Thinopyrum intermedium	**9**	**8**	**2.64**	**2.64**	**23**	**18**	**28**	**41**	**3.5**	**3.5**
Lupinus spp.	10	11	2.64	2.49	3	6	108	94	3.5	2.5
Potentilla gracilis	11	13	2.58	2.40	9	12	91	79	3.1	2.0
Achillea millefolium	12	10	2.56	2.52	6	5	101	99	2.9	2.7
Koeleria macrantha	13	14	2.40	2.38	7	10	98	85	2.0	1.9
Bromus carinatus	14	12	2.26	2.42	18	14	47	64	1.5	2.1
Danthonia unispicata	15	16	2.18	2.34	14	16	52	48	1.2	1.7
Danthonia intermedia	16	22	2.15	2.02	19	24	46	30	1.1	0.8
Phleum pratense	**17**	**19**	**2.10**	**2.15**	**27**	**26**	**22**	**22**	**1.0**	**1.2**
Poa compressa	**18**	**23**	**2.03**	**1.88**	**24**	**22**	**26**	**35**	**0.9**	**0.6**
Achnatherum occidentale	19	17	2.00	2.29	25	25	25	23	0.8	1.6
Veronica arvensis	**20**	**15**	**1.99**	**2.36**	**17**	**11**	**47**	**81**	**0.8**	**1.8**
Eriogonum spp.	21	20	1.97	2.11	13	15	54	52	0.7	1.0
Balsamorhiza incana	22	25	1.95	1.74	20	20	42	36	0.7	0.4
Bromus inermis	**23**	**24**	**1.94**	**1.79**	**28**	**28**	**10**	**12**	**0.7**	**0.5**
Geranium viscosissimum	24	27	1.88	1.64	22	27	32	18	0.6	0.4
Phlox spp.	25	26	1.88	1.68	15	21	50	35	0.6	0.4
Bromus tectorum	**26**	**18**	**1.86**	**2.24**	**26**	**17**	**24**	**46**	**0.6**	**1.4**
Hieracium cynoglossoides	27	29	1.79	1.59	16	23	48	31	0.5	0.3
Poa bulbosa	**28**	**30**	**1.73**	**1.08**	**31**	**31**	**3**	**4**	**0.4**	**0.1**
Epilobium brachycarpum	29	28	1.59	1.62	21	19	41	40	0.3	0.3
Festuca rubra	**30**	**21**	**1.55**	**2.05**	**30**	**30**	**5**	**7**	**0.3**	**0.9**
Solidago canadensis	31	31	1.00	0.90	29	29	8	10	0.1	0.1

Table 2: Non-native relative cover including difference (Time period 2 – Time period 1) and 95% confidence intervals for all plots combined (All) and each vegetation group defined from cluster analysis of species composition for time period 1(2008/2009).

	Non-native Relative Cover (%)		
Vegetation Group	2008/2009	2015/2016	Difference (CIs)
All	27.10	30.23	**3.13 (0.72, 5.73)**
Old Fields	68.08	60.98	-7.09 (-15.51, 2.98)
Mesic Prairie	16.23	22.33	**6.11 (3.40, 8.46)**
Xeric Prairie	32.98	33.98	1.00 (-3.17, 5.70)

Table 3: MRBP comparing vascular plant composition (plots = blocks), between two time periods (2008/2009 and 2015/2016) seven years apart (time period = groups).

**Vegetation Group 1 – Old Fields**							
	δ Under null hypothesis						
Distance Measure	Observed δ	Expected δ	Variance	Skewness	*T*	*A*	*P*
Sorensen	1.48	1.60	0.36 e-03	-1.04	-6.26	0.07	<<0.001
**Vegetation Group 2 – Mesic Prairie**							
	δ Under null hypothesis						
Distance Measure	Observed δ	Expected δ	Variance	Skewness	*T*	*A*	*P*
Sorensen	1.25	1.32	0.82 e-05	-1.02	-24.4	0.05	<<0.001
**Vegetation Group 3 – Xeric Prairie**							
	δ Under null hypothesis						
Distance Measure	Observed δ	Expected δ	Variance	Skewness	*T*	*A*	*P*
Sorensen	1.14	1.18	0.46 e-04	-0.88	-5.55	0.03	<<0.001

Table 4: Indicator species analysis results showing changes in relative cover and frequency (based on proportional abundance of a particular species in a given group relative to that species in all groups; groups being time periods), group assignment and indicator values (IV) for species with the strongest IVs and associated time period tendency for each community. Time period 1 was 2008 & 2009; time period 2 was 20015 & 2016. Tendency towards time period 1 indicates decreased abundance over time, and tendency towards time period 2 indicates increased abundance over time.

Species	Rel.Cover δ (%)	Rel.Freq δ (%)	Time period	IV	p-value
**Group 1: Old Fields**					
Poa compressa	-58	-43	1	63	**0.005**
Achillea millifolium	-26	-18	1	49.9	0.148
Poa pratensis	-18	-7	1	49.9	0.125
Lupinus spp.	-32	-13	1	39.4	0.127
Geum triflorum	+40	+12	2	51.7	0.124
Ventenata dubia	+70	+25	2	57.6	**0.020**
Festuca idahoensis	+46	+6	2	62.7	**0.001**
**Group 2: Mesic Prairie**					
Lupinus spp.	-26	-13	1	59.9	**0.0004**
Festuca idahoensis	-4	-1	1	51.2	0.119
Pseudoroegneria spicata	-2	-7	1	51.1	0.142
Poa secunda	0	-6	1	49.7	0.170
Arnica sorriea	-16	-3	1	48.8	0.186
Potentilla gracilis	-22	-11	1	48	**0.003**
Koeleria macrantha	-12	-8	1	47.9	0.151
Hieracium	-36	-22	1	35.5	**0.001**
Danthonia intermedium	-28	-11	1	22.9	**0.065**
Geranium viscossisimum	-32	-12	1	22.2	**0.023**
Balsomhorriza incana	-24	-9	1	22.1	0.113
Phlox spp.	-24	-7	1	22.1	0.119
Poa compressa	+32	+12	2	17.1	0.083
Thinopyrum intermedium	+38	+16	2	24.0	**0.014**
Veronica peregrina	+52	+38	2	51.7	**0.0002**
Ventenata dubia	+60	+26	2	61.0	**0.0002**
**Group 3: Xeric Prairie**					
Bromus annual spp.	-40	-23	1	63.7	**0.0002**
Arnica sorriea	-30	-25	1	50.5	**0.014**
Koeleria macrantha	-26	-26	1	47.1	0.062
Phlox spp.	-36	-26	1	45.5	**0.038**
Potentilla gracilis	-24	-10	1	31.6	0.118
Danthonia intermedium	-46	-22	1	26.1	0.098
Bromus carinatus	+62	+29	2	33.8	**0.013**
Poa compressa	+66	+23	2	24.3	**0.039**
Ventenata dubia	+10	0	2	52.5	0.188

Table 5: Quantile regression summary output including response and predictor variables, time, i.e., Time period (1 indicates the 2008/2009 time period; 2 indicates the 2015/2016 time period), slope coefficient and lower and upper bounds of the 95% confidence intervals. Significant (alpha = 0.05) slope coefficients are indicated in bold.

Response	Predictor	Time	Quantile	Slope Coeff	Lower bd	Upper bd
Native perennial cover	*V. dubia* cover	**1**	**0.5**	**-0.8**	**-1.21**	**-0.59**
		**1**	**0.9**	**-1.34**	**-1.92**	**-0.59**
		**2**	**0.5**	**-0.67**	**-0.79**	**-0.29**
		**2**	**0.9**	**-0.71**	**-0.86**	**-0.47**
Native perennial forb cover	*V. dubia* cover	**1**	**0.5**	**-0.61**	**-0.67**	**-0.52**
		**1**	**0.9**	**-1.26**	**-1.35**	**-0.99**
		**2**	**0.5**	**-0.22**	**-0.5**	**-0.14**
		**2**	**0.9**	**-0.39**	**-0.44**	**-0.25**
Native perennial grass cover	*V. dubia* cover	1	0.5	0.15	-0.29	0.63
		**1**	**0.9**	**-0.38**	**-0.63**	**-0.05**
		**2**	**0.5**	**-0.14**	**-0.37**	**-0.12**
		2	0.9	-0.25	-0.48	0.26
Other Non-native annual grass cover	*V. dubia* cover	**1**	**0.5**	**0.08**	**0.04**	**0.14**
		1	0.9	0.18	-0.01	0.70
		**2**	**0.5**	**0.09**	**0.01**	**0.12**
		2	0.9	0.04	-0.03	0.56
Non-native perennial grass cover	*V. dubia* cover	**1**	**0.5**	**-0.18**	**-0.30**	**-0.11**
		**1**	**0.9**	**-1.25**	**-1.44**	**-1.05**
		**2**	**0.5**	**-0.09**	**-0.18**	**-0.05**
		**2**	**0.9**	**-0.05**	**-0.72**	**-0.01**
